# Characteristics and Outcomes of Young Patients with First-Ever Ischemic Stroke Compared to Older Patients: The National Acute Stroke ISraeli Registry

**DOI:** 10.3389/fneur.2017.00421

**Published:** 2017-08-21

**Authors:** Miri Lutski, Inbar Zucker, Tamy Shohat, David Tanne

**Affiliations:** ^1^The Israel Center for Disease Control, Ministry of Health, Ramat Gan, Israel; ^2^Department of Epidemiology and Preventive Medicine, School of Public Health, Sackler Faculty of Medicine, Tel Aviv University, Tel-Aviv, Israel; ^3^The Sagol Neuroscience Center, Sheba Medical Center, Tel-Hashomer, Israel

**Keywords:** adults under age 50 years, first-ever ischemic stroke, epidemiology of stroke, outcomes of stroke, stroke, stroke registry

## Abstract

**Background:**

Nationwide data on the clinical profile and outcomes of ischemic stroke in younger adults are still scarce. Our aim was to analyze clinical characteristics and outcomes of young patients with first-ever ischemic stroke compared to older patients.

**Methods:**

The National Acute Stroke ISraeli registry is a nationwide prospective hospital-based study performed triennially. Younger adults, aged 50 years and younger, were compared with patients, aged 51–84 years regarding risk factors, clinical presentation, stroke severity, stroke etiology, and outcomes. A logistic model for stroke outcome was fitted for each age group.

**Results:**

336 first-ever ischemic strokes were identified among patients aged 50 years and younger and 3,243 among patients 51–84 years. Younger adults had lower rates of traditional vascular risk factors, but 82.7% had at least one of these risk factors. Younger adults were more likely to be male (62.8%), current smokers (47.3%), and to have a family history of stroke (7.4%). They tended to have less common stroke presentation such as sensory disturbances or headache and were more likely to arrive at the hospital independently by car. The majority of young adults (70%) had a favorable outcome (modified Ranking Scale; mRS ≤ 1) at discharge, but 11.7% had poor outcome (mRS > 3) and 18.2% had an in-hospital complication. According to a multivariable regression model, in young adults, only baseline stroke severity (National Institute of Health Stroke Scale > 5) was associated with poor outcome at discharge (*p* < 0.001), whereas in older adults, stroke severity (*p* < 0.001), female gender (OR = 1.35, CI 95% 1.03–1.76), older age (OR = 1.08, CI 95% 1.01–1.16), atrial fibrillation (OR = 1.62, CI 95% 1.16–2.26), and anterior circulation territory (OR = 2.10, CI 95% 1.50–2.94) were all significantly associated with poor outcome.

**Conclusion:**

Our findings, in this nationwide registry, demonstrate the relatively high rate of smoking and family history of stroke, and the lower rate of hospital arrival by ambulance among young adults. This calls for increasing awareness to the possibility of stroke among young adults and for better prevention, especially smoking cessation.

## Introduction

Stroke in younger adults is less frequent than in older people; however, its health and economic impact on younger individuals, their family, and society is major (1). The incidence rates of ischemic stroke in younger adults vary from 6.6 to 11.4 in 100,000 people per year (1). Incidence rates of ischemic stroke have increased in adults aged 55 years and under in the United States (2) and in Europe (3, 4). This trend could reflect changes in the burden of classical vascular risk factors in younger individuals, such as increases in prevalences of diabetes, obesity, and hypercholesterolemia or result from the interplay of other risk factors (3).

Associations between risk factors for acute ischemic stroke and clinical outcomes have been analyzed predominantly in older rather than younger patients (5–10). However, the knowledge gleaned from research of older adults cannot always be applied to younger adults (11, 12). Previous studies showed that both younger and older age stroke patients shared the same modifiable risk factors (11). However, the prevalence of these risk factors was different in these two age groups (1, 11). Risk factors for atherosclerosis such as dyslipidemia, hypertension, and diabetes were less frequent among young stroke patients aged 40 and under, whereas other risk factors such as family history, coagulation disorders, patent foramen ovale and non-atherosclerotic vasculopathies were more frequent (1, 13).

A previous study from the National Acute Stroke ISraeli (NASIS) registry found major differences between the clinical characteristics and outcomes of the very elderly (aged ≥85 years) patients with first-ever ischemic stroke (14); therefore, the comparison group used in this study was defined as patients aged 51–84. The purpose of our study was to examine risk factors, clinical characteristics, and in-hospital outcomes of younger adults (aged ≤50 years) compared to patients 51–84 years old, with first-ever ischemic stroke in the NASIS registry.

## Materials and Methods

### Study Design and Population

National Acute Stroke ISraeli is a prospective hospital-based nationwide registry performed triennially (15). Data were collected prospectively by coordinating physicians for all consecutive cases of acute cerebrovascular diseases hospitalized during 2-month periods in 2004, 2007, 2010, and 2013 at all general hospitals in Israel. The NASIS registry was approved by the ethical committees of all participating hospitals.

### Data Collection

The registry was previously described in detail (15). In brief, for the NASIS registry, a specially designed structured form was used to collect data, including demographics, risk factors, comorbidities, stroke characteristics, in-hospital management, and in-hospital outcome. Stroke severity was determined using the National Institute of Health Stroke Scale (NIHSS) score (16). The modified Ranking Scale (mRS) (17) was used for assessing disability before stroke and at discharge. The TOAST classification was used to define the etiology of stroke based on in-hospital investigation (18). Prior atherosclerosis was defined as prior TIA, carotid stenosis, peripheral artery disease, or ischemic heart disease. Modifiable vascular risk factors included diabetes, dyslipidemia, smoking, hypertension, and obesity. Participants were classified into four groups: no risk factors, one, two, and three or more modifiable risk factors.

### Outcome Variables

Two outcomes were studied. Poor outcome—defined as severe disability or death (mRS > 3) at discharge; and complications during hospitalization, categorized to neurological, cardiac, infectious, and other.

### Statistical Analysis

Younger adults, aged 50 years and younger, were compared with patients, aged 51–84 years regarding their baseline characteristics, stroke severity at admission, stroke etiology, and stroke outcomes.

Chi-square test was used to determine the differences between groups in the categorical variables. Continuous variables were compared using the *T*-test or the Mann–Whitney test. Univariate and multivariable logistic regression models were fitted separately for each age group (aged 50 years and younger and 51–84 years) to identify covariates associated with poor outcome. The registry period, age, and gender were entered in all multivariable models. Other predictors that had a level of significance of less than 0.1 in the univariate analysis were included in the final multivariable logistic regression models. Additionally, we tested for potential interactions between the predictors and no significant interactions were found. We excluded from the logistic regression models patients with prior disability (mRS ≥ 2) and patients with length of hospitalization of more than 1 month (approximately 1.6% among younger adults and 3.1% among patients 51–84 years old). The analyses were performed using SPSS 23 software (SPSS Inc., Chicago, IL, USA).

## Results

### Patient Characteristics

**Figure 1:**
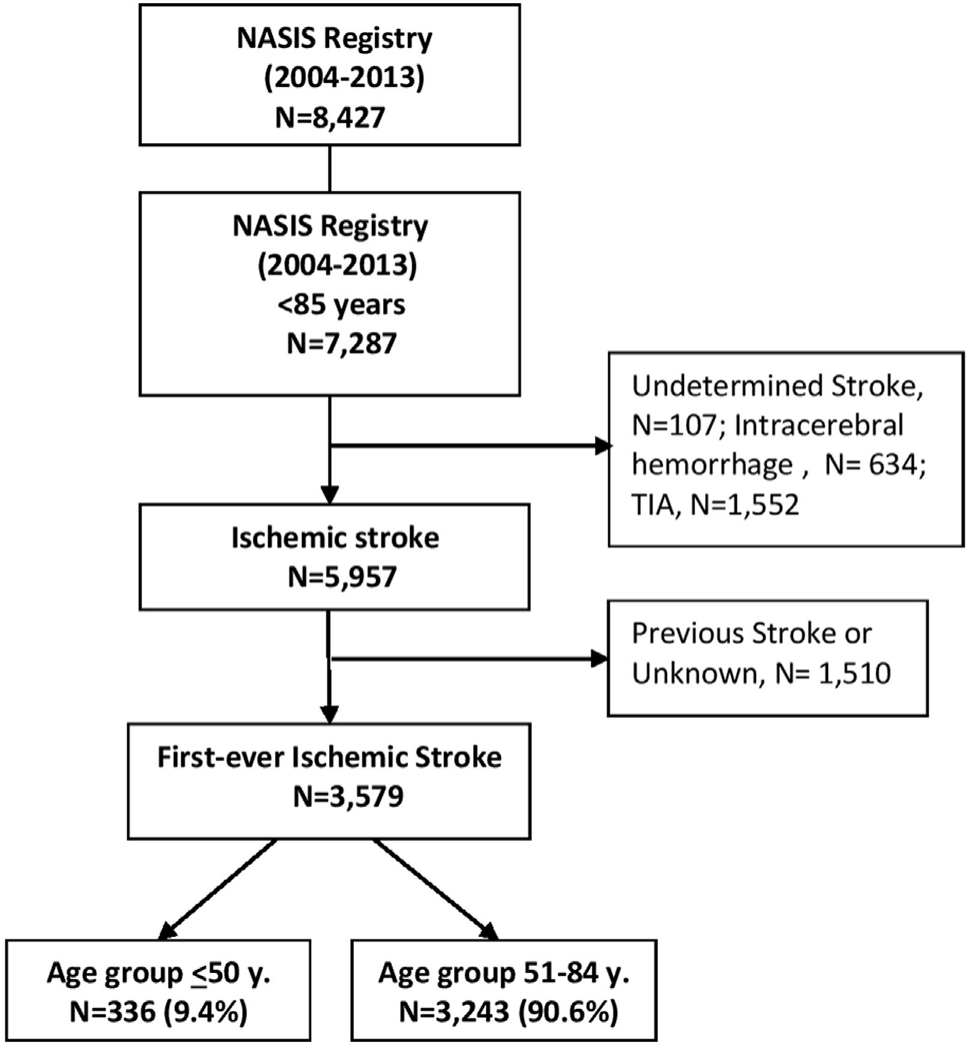
Study flowchart.

A total of 336 (9.4%) first ever ischemic strokes were identified among patients aged 50 years and younger and 3,243 among patients 51–84 years (Figure 1).

Characteristics and health-related conditions of the patients, by age group, are presented in Table 1. Male gender and Arab ethnicity were significantly associated with the younger age group. Younger adults were more likely to be current smokers than were older patients (47.3 vs. 21.9%) and were more likely to have a first degree relative who had a stroke before the age of 55 years (7.4 vs. 2.0%). The younger group comprised a higher proportion of patients without vascular risk factors (17.4 vs. 5.9%) and a lower proportion with three or more vascular risk factors (26.7 vs. 41.9%). However, a total of 82.7% had at least one modifiable vascular risk factor.

**Table 1:** Characteristics and health-related conditions of stroke patients, by age group.

	Age groups (years)		*p* Value
	≤50 *N* = 336 *n* (%)	51–84 *N* = 3,243 *n* (%)	
**Demographic data**			
Gender			
Male	211 (62.8)	1,833 (56.5)	0.03
Female	125 (37.2)	1,410 (43.5)	
Population group			
Jews	232 (73.2)	2,605 (84.8)	<0.001
Arab	71 (22.4)	398 (13.0)	
Others	14 (4.4)	68 (2.2)	
**Known risk factors and comorbidities**			
Current smoking	159 (47.3)	702 (21.9)	<0.001
Hypertension	133 (39.7)	2,499 (77.3)	<0.001
Diabetes	76 (22.6)	1,410 (43.5)	<0.001
Dyslipidemia	161 (48.2)	2,007 (62.2)	<0.001
Obesity	73 (22.5)	644 (20.9)	0.5
Atrial fibrillation	9 (2.7)	548 (17.0)	<0.001
Congestive heart failure	12 (3.6)	391 (12.1)	<0.001
Chronic kidney disease	14 (4.2)	377 (11.7)	<0.001
Peripheral artery disease	7 (2.1)	191 (5.9)	0.004
Prior TIA	(3.6) 12	201 (6.3)	0.05
Known carotid stenosis >50%	2 (0.6)	79 (2.5)	0.03
Ischemic heart disease	33 (9.8)	891 (27.5)	<0.001
Family history of stroke	24 (7.4)	61 (2.0)	<0.001
APLS	6 (1.8)	12 (0.4)	<0.001
Known patent foramen ovale	14 (11.3)	18 (2.8)	<0.001
Prior disability (modified Ranking Scale ≥ 2)	12 (3.6)	612 (19.3)	<0.001
Score of modifiable vascular risk factors															
No	56 (17.4)	181 (5.9)	<0.001
1	90 (28.0)	628 (20.6)	
2	90 (28.0)	961 (31.6)	
3+	86 (26.7)	1,275 (41.9)	
Prior atherosclerosis			
No	283 (85.2)	2,053 (64.5)	<0.001
Yes	49 (14.8)	1,129 (35.5)	
**Medications prior to event**			
Statin	72 (21.8)	1,389 (44.0)	<0.001
ACE/ARB	61 (18.5)	1,405 (44.3)	<0.001
Antiplatelet	67 (20.1)	1,513 (47.8)	<0.001
Anticoagulants	15 (4.5)	276 (8.7)	0.009
**Mode of arrival to emergency room**			
Ambulance	102 (32.2)	1,454 (47.6)	<0.001
Private car	183 (57.7)	1,379 (45.2)	
Transfer from other hospital	17 (5.4)	43 (1.4)	
Other	15 (4.7)	177 (5.8)	
**Time delay (h) from stroke onset to ER arrival and ER-CT^a^ (mean ± SD)**			
Stroke onset-ER time	5.55 ± 5.48	5.51 ± 5.54	0.72
ER-CT time	3.53 ± 4.48	3.39 ± 4.53	0.63
**Revascularization**			
Thrombolysis	17 (5.1)	159 (4.9)	0.89
Mechanical revascularization	8 (11.1)	24 (3.0)	<0.001

*ACE/ARB, angiotensin-converting-enzyme inhibitor/angiotensin II receptor antagonists*.

*^a^Data missing for approximately 30% of cases. Patients with in-hospital events and those transferred from other hospitals excluded from analysis*.

Stroke characteristics by age group categories are presented in Table 2. The distribution of stroke etiology was significantly different between the two groups. Younger adults had a smaller proportion of cardioembolic strokes (8.0 vs. 12.0%) and of occlusive disease strokes (17.6 vs. 24.0%), while strokes due to artery dissection and/or hematological disorder were more common in this group (6.0 vs. 0.5%). The two age groups also differed in the clinical classification of strokes and its severity (Table 2).

**Table 2:** Stroke characteristics, severity at admission, by age group.

	Age groups (years)		*p* Value
	≤50 *N* = 336 *n* (%)	51–84 *N* = 3,243 *n* (%)	
**Stroke etiology**			
Cardioembolic	27 (8.0)	390 (12.0)	<0.001
Large vessel occlusive disease	22 (6.5)	216 (6.7)	
Small vessel occlusive disease	59 (17.6)	777 (24.0)	
Other^a^	20 (6.0)	17 (0.5)	
Undetermined	203 (60.4)	1,815 (56.0)	
Procedure related	5 (1.5)	28 (0.9)	
**Clinical classification of ischemic stroke, *n* (%)**			
Lacunar	107 (31.8)	846 (26.2)	0.02
Total anterior circulation	17 (5.1)	235 (7.3)	
Partial anterior circulation	115 (34.2)	1,234 (38.2)	
Posterior circulation	71 (21.1)	749 (23.2)	
Unknown	26 (7.7)	163 (5.1)	
**Stroke severity, *n* (%)**			
National Institute of Health Stroke Scale															
≤5	242 (72.2)	1,933 (59.7)	<0.001
6–10	54 (16.1)	737 (22.8)	
11–15	15 (4.5)	261 (8.1)	
16–20	9 (2.7)	185 (5.7)	
>20	15 (4.5)	121 (3.7)	
**Length of hospitalization**			
Length (days)	6.11 ± 5.1	6.6 ± 5.6	0.054

*^a^Cervical artery dissection, hematological disorder (hypercoagulability, antiphospholipid antibody syndrome, etc.)*.

**Figure 2:**
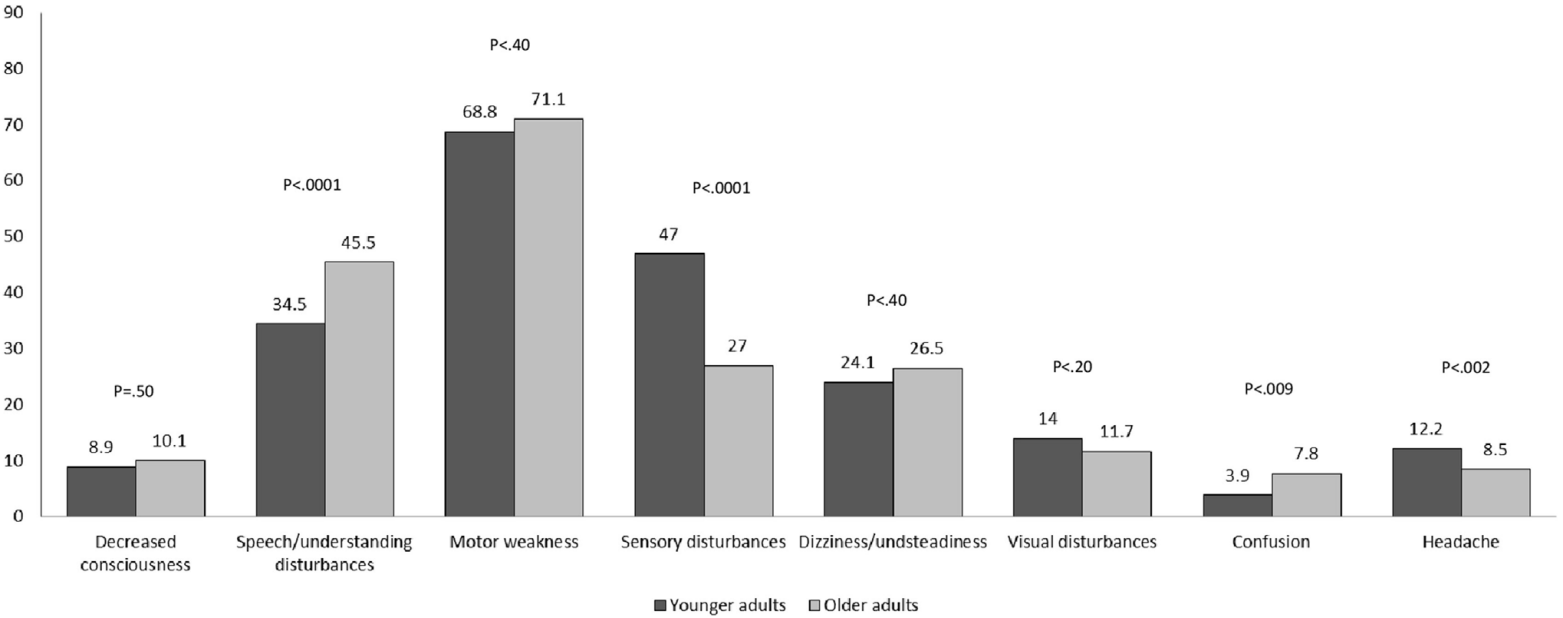
Prevalence of presenting symptoms by age group (%).

The presenting symptoms of stroke by age groups are depicted in Figure 2. Motor weakness was the most frequent disturbance in both age groups. Sensory disturbances and headache were more common among younger than older patients, while speech disturbances were less common.

**Figure 3:**
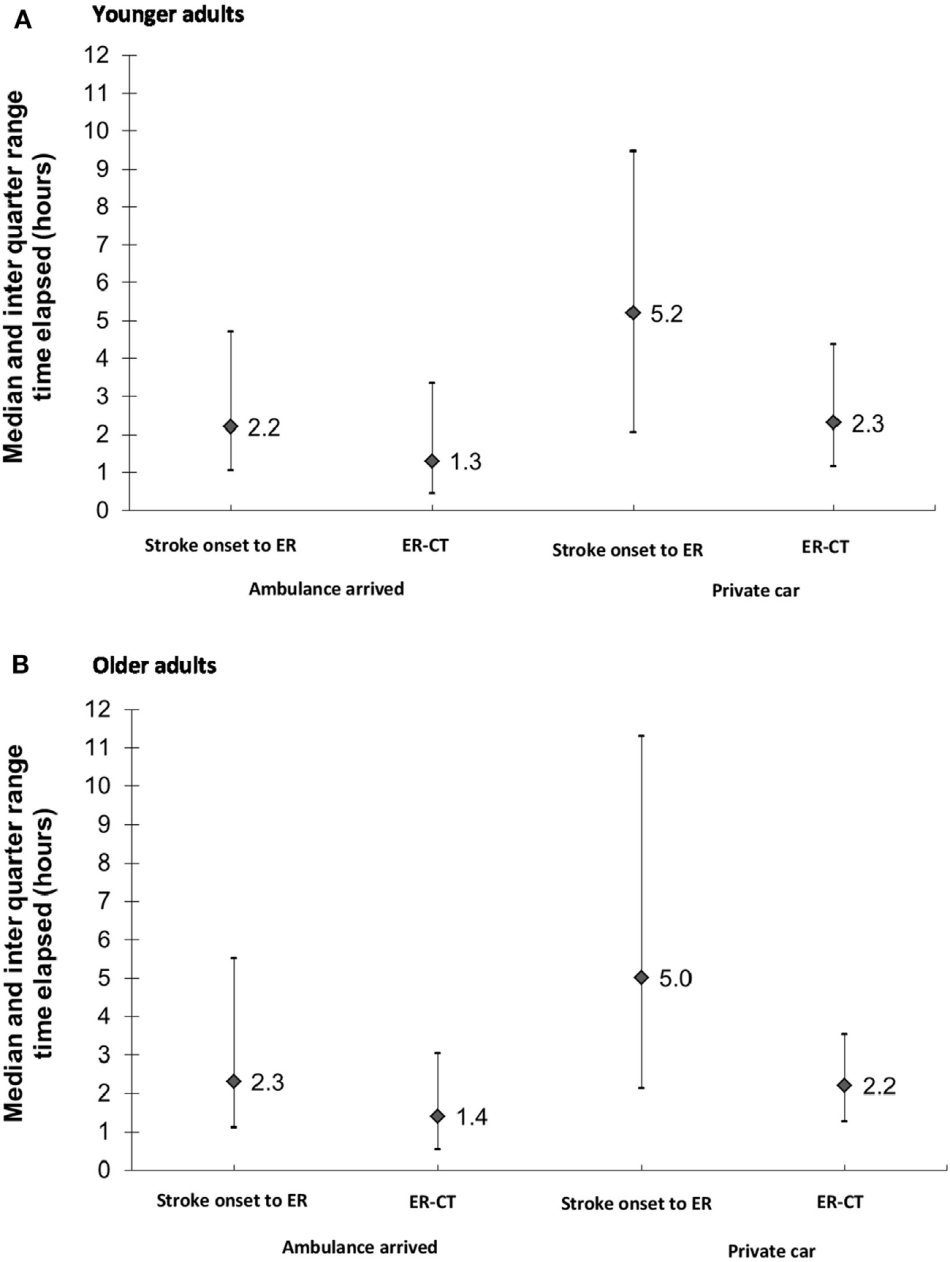
**(A,B)** Time elapsed* (h) from stroke onset to ER arrival** and ER-CT by transportation arrived. **(A)** Younger adults and **(B)** older adults. *Data missing for approximately 30% of cases; **Patients with in-hospital events and those transferred from other hospitals were excluded from the analysis.

Only one-third (32%) of younger adults arrived by ambulance compared to almost half (48%) of the older patients (Table 1). Among younger patients with non-minor strokes (baseline NIHSS > 5) only 61.0% arrived by ambulance compared to 71.4% of older patients (data not shown). In both age groups, the median time elapsing from the onset of symptoms to hospital arrival (onset-to-door) and the median time elapsing from hospital arrival to undergoing CT (door-to-CT) were shorter in patients who arrived by ambulance compared to patients who arrived independently (2.2 h compared to 5.2 h; 1.3 and 2.3 h, respectively) (Figures 3A,B).

### Stroke Outcome at Discharge

**Figure 4:**
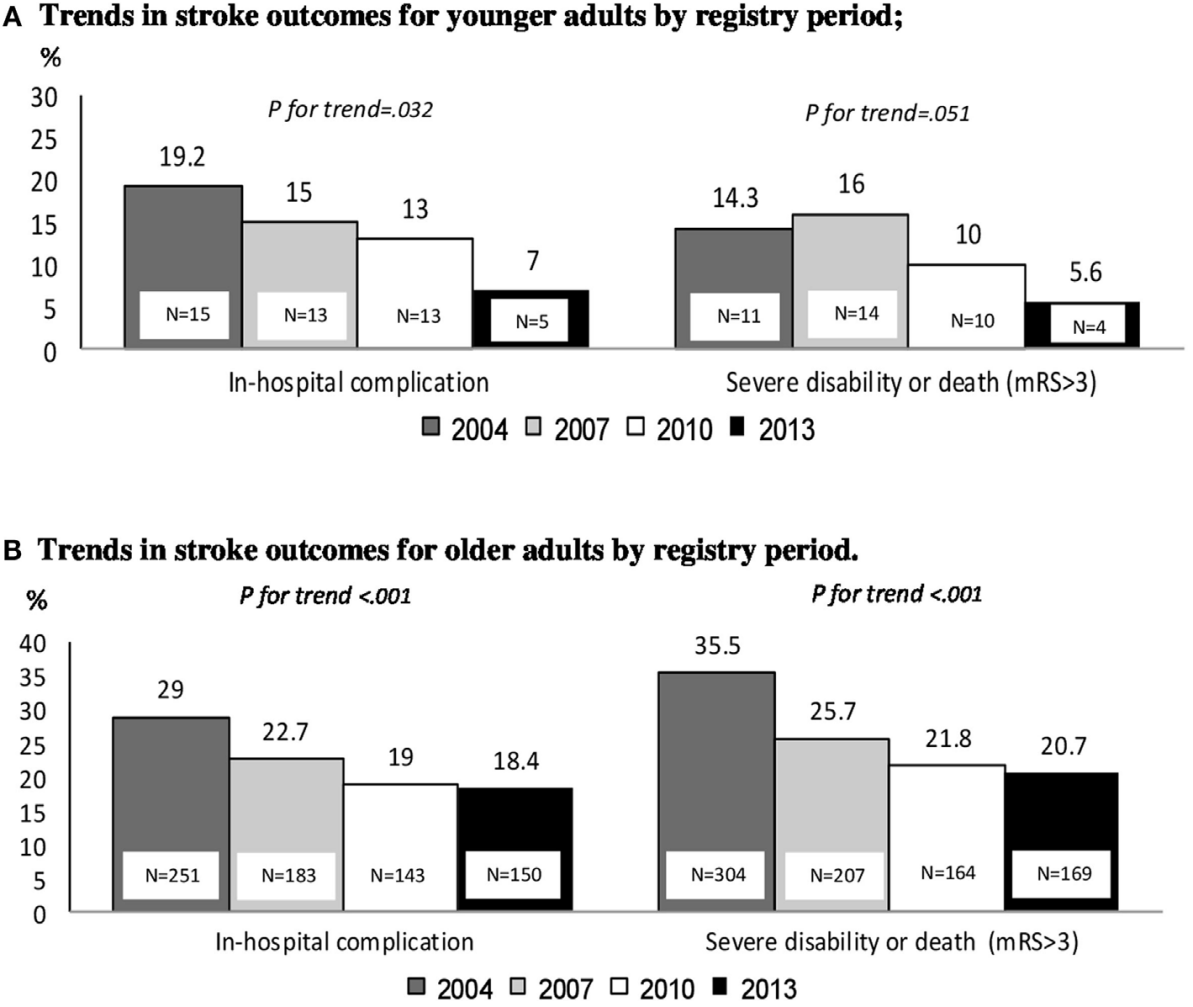
**(A,B)** Trends in stroke outcomes by registry period. **(A)** Trends in stroke outcomes for younger adults by registry period and **(B)** trends in stroke outcomes for older adults by registry period.

Stroke outcome was more favorable among younger adults (70%), with lower rates of poor outcome (mRS > 3) at discharge (11.7%) and lower rates of in hospital complications (18.2%) compared to older patients (Table 3). Over the four registry periods, the rates of severe disability or death (mRS > 3) declined among both groups, from 14.3% in 2004 to 5.6% in 2013 (*p* for trend = 0.05) among younger adults and from 35.5% in 2004 to 20.7% in 2013 (*p* for trend <0.001) among older patients (Figures 4A,B). No significant decline was found in stroke severity distribution during the 9-year registry period among young adults (*p* for trend = 0.18), whereas among older patient, there was a significant decline in severity NIHSS > 5 during the 9-year registry period with *p* for trend <0.001 (data not shown).

**Table 3:** Outcomes of first ischemic stroke by age groups.

	Age groups (years)		*p* Value
	≤50 *N* = 336	51–84 *N* = 3,243	
**In-hospital complications, *n* (%)**			
Neurological	19 (5.7)	282 (8.7)	0.06
Cardiac	4 (1.2)	104 (3.2)	0.04
Infectious	27 (8.0)	403 (12.4)	0.02
Bleeding	2 (0.6)	53 (1.6)	0.04
Other	9 (2.7)	142 (4.4)	0.10
**Disability at discharge and in-hospital mortality, *n* (%)**
Modified Ranking Scale at discharge			
0–1	235 (70.1)	1,370 (42.5)	<0.001
2–3	61 (18.2)	1,013 (31.4)	
4–5	31 (9.3)	694 (21.5)	
In-hospital death	8 (2.4)	150 (4.6)	

Multivariable logistic regression models for poor outcome were fitted separately for each age group (Table 4). The variables adjusted for in these final models were chosen according to univariate models in Table S1 in Supplementary Material. According to multivariable regression models, stroke severity (NIHSS > 5) at admission was positively associated with poor outcome (mRS > 3) in both age groups (Table 4). In older patients, older age (OR = 1.08, CI 95% 1.01–1.16), female gender (OR = 1.35, CI 95% 1.03–1.76), atrial fibrillation (OR = 1.62, CI 95% 1.16–2.26), and anterior circulation territory (OR = 2.10, CI 95% 1.50–2.94) were also associated with poor outcome. In younger adults, female gender (OR = 2.37, CI 95% 0.89–6.33), posterior circulation territory (OR = 4.25, CI 95% 0.93–19.42), and prior atherosclerosis (OR = 2.89, CI 95% 0.91–9.18) were associated with poor outcome; however, these associations did not reach statistical significance (Table 4).

**Table 4:** Independent predictors of severe disability (modified Ranking Scale > 3) or in-hospital mortality by age group.

		18–50 years		51–84 years	
		OR (95% CI)	*p*-Value	OR (95% CI)	*p*-Value
Age (for every increase in 5 years)		0.94 (0.67–1.33)	0.73	1.08 (1.01–1.16)	0.03
Female gender		2.37 (0.89–6.33)	0.09	1.35 (1.03–1.76)	0.03
National Institute of Health Stroke Scale at admission	>5	27.38 (7.79–93.15)	<0.001	13.04 (9.67–17.60)	<0.001
	≤5	ref		ref	
Clinical subtypes of stroke	TAC/PAC	3.03 (0.75–12.16)	0.12	2.10 (1.50–2.94)	<0.001
	PC	4.25 (0.93–19.42)	0.06	1.08 (0.71–1.65)	0.7
	Lacunar	ref		ref	
Prior atherosclerosis		2.89 (0.91–9.18)	0.07	1.31 (0.99–1.73)	0.06
Atrial fibrillation		–		1.62 (1.16–2.26)	0.005
Previous statin use		–		0.75 (0.55–1.02)	0.07
Obesity		–		1.18 (0.87–1.61)	0.28
Dyslipidemia		–		1.20 (0.88–1.63)	0.25

*The models were adjusted for the registry period*.

*TAC/PAC, total anterior circulation/partial anterior circulation; PC, posterior circulation*.

## Discussion

In this nationwide study, nearly half of younger adults with first-ever ischemic stroke reported current smoking. This is considerably higher than the reported prevalence of smoking among the general Israeli population aged 21–50 (21.2%) (19). The relatively high rate of smoking among younger stroke patients highlights its significance as an important modifiable risk factor for stroke in this age group and emphasizes the need for better smoking prevention strategies that address younger adults. Similar to previous studies (20, 21), ischemic stroke among younger patients was more common among males.

The majority of stroke cases in younger adults were related to the existence of traditional stroke risk factors, though the prevalence of these factors was lower than in older patients; concurring with other studies (7). Two or more vascular risk factors in young adults were found in 55% of our patients, which was similar to the 52% reported in the registries of Zurich and Bern (9). This highlights the importance of these modifiable risk factors for the occurrence of stroke in all ages. Therefore, targeting these risk factors is essential for primary prevention of stroke. However, 17% of the young adults in our study had none of the classical risk factors. In these cases, other potential risk factors such as genetic coagulation disorders should be considered. Indeed, younger adults were more likely to have a family history of stroke than were older patients. This calls for increased awareness toward appropriate review of family history in younger adults.

Notably, among younger compared to older patients, first ischemic strokes were more likely to be caused by such etiologies as cervical artery dissection and hematologic disorders, and less likely by cardioembolic source. In line with previous studies, we found that in most cases of ischemic stroke in younger adults, the underlying etiology remained unclear (1). Similarly, in patients aged 15–49 years with first-ever ischemic stroke from 15 European stroke centers, the etiology remained undetermined in 40% (7). A reason for our particularly high proportion of undetermined stroke is that subtyping of stroke etiology was based only on information available at hospital discharge. In practice, the work-up of stroke etiology is often done following discharge by diagnostic tests performed in the community.

Younger adults had a high proportion of lacunar strokes, whereas stroke of the anterior circulation was more common among older patients. This partially explains the higher proportion of mild stroke (NIHSS ≤ 5) among younger adults in this study as lacunar strokes are known to be less severe (6, 9, 22).

Younger adults with stroke were more likely to arrive to the hospital by private car than were older patients. This finding is of interest since according to our data, arrival by car was associated with longer onset-to door and door-to-CT times. Provision of timely treatment in acute stroke is critical, especially in the current era of stroke care, with the advancement of reperfusion strategies by thrombolysis and mechanical thrombectomy (21). Not only is early hospital arrival in acute stroke critical to reperfusion therapy but it also increases the probability of survival and affects other clinical outcomes in all patients with ischemic stroke, even when reperfusion therapy is not used (23). Possibly, the delay in hospital arrival of young adults is due to less common stroke presentation, as reported here. In addition, younger adults may have lower awareness to the possibility of stroke. In this study, motor weakness was the most frequent disturbance in both age groups; however, the proportions with sensory disturbances and headaches were higher among the younger patients. Indeed, in our study, patients with sensory disturbances or headaches were more likely to arrive by private car than were patients presenting with motor weakness, decreased consciousness, or speech disturbances.

Another important finding of this study is the substantial decline in poor stroke outcome (mRS > 3) among both age groups. A decline in stroke severity (NIHSS > 5) during the 9-year registry period was observed in both age group; however, only among older patients it reached statistical significance. This can be explained by improved management of known vascular risk factors such as hypertension, dyslipidemia, and diabetes mellitus (24). In addition, the growing use of reperfusion modalities may have also contributed to the improved outcomes. It should be noted that in the registry periods the use of reperfusion therapy was still limited but has been expected to grow since, especially following the publications of landmark studies that established the efficacy of mechanical revascularization (25, 26). The decline in the overall complication rate in both age groups during the 9-year registry period can be at least partially explained by improved acute stroke care (27). Of the factors investigated in this study, only the severity of stroke at admission was positively associated with poor outcome in younger adults. In the Zurich and Bern ischemic stroke registries, stroke of the anterior circulation, older age, and severity of stroke were associated with unfavorable outcome or death at 3 months (9), while in the Swiss Young Stroke Study, severity of stroke and diabetes were reported to predict unfavorable outcome at 3 months (8). This difference might be explained by the fact that previous studies focused on clinical outcomes at 3 months after the ischemic stroke (8, 9), while this study focused only on in-hospital outcomes. Yet, prior atherosclerosis, female gender, and posterior circulation territory were found to be associated with unfavorable clinical outcome among younger adults with borderline significance; this may be due to insufficient statistical power.

Our study has several methodological strengths, including its good external validity since data were obtained from a non-selective nationwide registry. In addition, our study used a validated measure of functioning, which was consistently measured at admission and at discharge. Some methodological limitations, however, should be considered. A major limitation of our study is the extremely high rate of stroke of undetermined origin, because the data collected were limited to the hospitalization period. In Israel, diagnostic tests aimed at identifying the stroke etiology (such as coagulation and genetic tests, echocardiography, Holter ECG) is often performed in the community. Yet, other studies with the exception of three studies (9, 10, 28) showed that despite a broad diagnostic approach and availability of accurate diagnostic tools, the proportion of stroke of undetermined etiology still remains high among young stroke patients (7, 11). Second, the study included only hospitalized stroke patients; and information on patients not admitted was not available. Nonetheless, there are no known barriers for hospital admission in the Israeli health-care system. Other limitation is the absence of data on recreational drug and oral contraceptive; however, recreational drugs are a rare risk factor for stroke in young Western populations (13), and the role of oral contraceptives as a risk factor for ischemic stroke remains still controversial (12). Another limitation is the absence of data on neuroimaging features such as ischemic stroke volume and previous small vessel disease, which can affect stroke outcome. It is well known that small-vessel disease can produce small deep hemispherical or brainstem lacunar infarcts in young adults, usually in patients with vascular risk factors (12).

In conclusion, our findings, in this nationwide registry, demonstrate that young adults have high prevalence of modifiable vascular risk factors and especially a high rate of smoking. In addition, our findings demonstrate a relatively high rate of family history of stroke, and lower rate of hospital arrival by ambulance among young adults. In our study, stroke in young patients was associated with poor outcome at discharge in almost 12%, and this may lead to major morbidity and long-term socioeconomic consequences. This calls for increasing awareness to the possibility of stroke among young adults and for better prevention, especially smoking cessation.

## Ethics Statement

The NASIS registry was approved by the ethical committees of all participating hospitals.

## Author Contributions

ML, IZ, TS, and DT contributed to the design, data interpretation, and critical review. DT contributed to the data collection. ML and IZ contributed to draft the manuscript and data analysis. All authors contributed to revising the manuscript for publication and approved the final version for publication.

## Conflict of Interest Statement

The authors declare that the research was conducted in the absence of any commercial or financial relationships that could be construed as a potential conflict of interest.
