# Supplementary material for: Characteristics and Outcomes of Young Patients with First-Ever Ischemic Stroke Compared to Older Patients: The National Acute Stroke ISraeli Registry
Source: Front Neurol. 2017 Aug 21;8:421. doi: 10.3389/fneur.2017.00421 (PMC5566555; doi:10.3389/fneur.2017.00421)
Supplement: Supplementary file 1 [file Table_1.DOCX]

**Supplemental table 1 Univariate analysis of independent predictors of poor outcome by age group**

|  |  | **18-50 years** | | **51-84 years** | |
| --- | --- | --- | --- | --- | --- |
|  |  | **OR (95% CI)** | **P** | **OR (95% CI)** | **P** |
| Age ( for every increase in 5 years) |  | 0.90 (0.69-1.17) | 0.43 | 1.23 (1.17-1.30) | **<0.001** |
| Female gender |  | 1.81 (0.87-3.78) | 0.11 | 1.50 (1.23-1.83) | **<0.001** |
| NIHSS at admission | >5 | 27.75 (9.36-82.32) | **<0.001** | 19.14 (14.55-25.18) | **<0.001** |
|  | ≤5 | ref |  | ref |  |
| Clinical subtypes of stroke | TAC/PAC | 5.94 (1.70-20.80) | **0.005** | 4.25 (3.21-5.63) | **<0.001** |
|  | PC | 5.61 (1.49-21.20) | **0.011** | 1.21 (0.85-1.73) | 0.29 |
|  | Lacunar | ref |  | ref |  |
| Prior atherosclerosis |  | 2.12 (0.89-5.07) | 0.09 | 1.23 (0.99-1.51) | 0.054 |
| Atrial fibrillation |  | * |  | 1.58 (1.15-2.17) | **0.005** |
| Previous statin use |  | 0.85 (0.33-2.15) | 0.73 | 0.73 (0.60-0.90) | **0.003** |
| ACE/ARB |  | 0.82 (0.30-2.23) | 0.70 | 0.98 (0.80-1.20) | 0.843 |
| Current smoking |  | 0.82 (0.39-1.72) | 0.60 | 0.89 (0.68-1.18) | 0.41 |
| Hypertension |  | 0.66 (0.30-1.46) | 0.31 | 1.10 (0.87-1.40) | 0.42 |
| Diabetes |  | 0.94 (0.39-2.28) | 0.89 | 1.08 (0.89-1.32) | 0.44 |
| Dyslipidemia |  | 0.75 (0.35-1.57) | 0.44 | 0.75 (0.61-0.92) | **0.005** |
| Obesity |  | 1.07 (0.44-2.58) | 0.89 | 0.78 (0.62-0.99) | **0.04** |
| Registry period |  | 0.65 (0.45-0.94) | **0.02** | 0.78 (0.711-0.85) | **<0.001** |

TAC/PAC= Total Anterior Circulation/ Partial Anterior Circulation, PC= Posterior Circulation; * n=9 patients.
